# Hyponatremia influences the outcome of patients with acute-on-chronic liver failure: an analysis of the CANONIC study

**DOI:** 10.1186/s13054-014-0700-0

**Published:** 2014-12-13

**Authors:** Andrés Cárdenas, Elsa Solà, Ezequiel Rodríguez, Rogelio Barreto, Isabel Graupera, Marco Pavesi, Faouzi Saliba, Tania Mara Welzel, Javier Martinez-Gonzalez, Thierry Gustot, Mauro Bernardi, Vicente Arroyo, Pere Ginès, on behalf of the CANONIC study investigators of the EASL-CLIF Consortium

**Affiliations:** GI Unit, Hospital Clinic, University of Barcelona, Carrer Villarroel, 170, 08036 Barcelona, Spain; Liver Unit, Hospital Clinic, University of Barcelona, Carrer Villarroel, 170, 08036 Barcelona, Spain; Data Management Center - CLIF Consortium, Hospital Clinic, Carrer Villarroel, 170, 08036 Barcelona, Spain; AP-HP Hôpital Paul Brousse Centre Hépato-Biliaire, University Paris-Sud, UMR-S 785, 12 avenue Paul Vaillant Couturier, 94800 Villejuif, France; Department of Medicine 1, JW Goethe University Hospital, Frankfurt Theodor-Stern-Kai 7, D-60590 Frankfurt am Main, Germany; Department of Gastroenterology and Hepatology, Hospital Universitario Ramón y Cajal, IRYCIS, Carretera. de Colmenar Viejo, km 9100, 28034 Madrid, Spain; Liver Unit, Department of Gastroenterology and Hepatopancreatology, Erasme University Hospital, Université Libre de Bruxelles, Route de Lennik 808, 1070 Brussels, Belgium; Department of Medical and Surgical Sciences, University of Bologna, Via Giuseppe Massarenti 9, 40138 Bologna, Italy; University of Barcelona, IDIBAPS, CIBEReHD, IRSIN, Carrer Villarroel, 170, 08036 Barcelona, Catalunya Spain

## Abstract

**Introduction:**

Hyponatremia is a marker of poor prognosis in patients with cirrhosis. This analysis aimed to assess if hyponatremia also has prognostic value in patients with acute-on-chronic liver failure (ACLF), a syndrome characterized by acute decompensation of cirrhosis, organ failure(s) and high short-term mortality.

**Methods:**

We performed an analysis of the Chronic Liver Failure Consortium CANONIC database in 1,341 consecutive patients admitted to 29 European centers with acute decompensation of cirrhosis (including ascites, gastrointestinal bleeding, hepatic encephalopathy, or bacterial infections, or any combination of these), both with and without associated ACLF (301 and 1,040 respectively).

**Results:**

Of the 301 patients with ACLF, 24.3% had hyponatremia at inclusion compared to 12.3% of 1,040 patients without ACLF (*P* <0.001). Model for end-stage liver disease, Child-Pugh and chronic liver failure-SOFA scores were significantly higher in patients with ACLF and hyponatremia compared to those without hyponatremia. The presence of hyponatremia (at inclusion or during hospitalization) was a predictive factor of survival both in patients with and without ACLF. The presence of hyponatremia and ACLF was found to have an independent effect on 90-day survival after adjusting for the potential confounders. Hyponatremia in non-ACLF patients nearly doubled the risk (hazard ratio (HR) 1.81 (1.33 to 2.47)) of dying at 90 days. However, when considering patients with both factors (ACLF and hyponatremia) the relative risk of dying at 90 days was significantly higher (HR 6.85 (3.85 to 12.19) than for patients without both factors. Patients with hyponatremia and ACLF had a three-month transplant-free survival of only 35.8% compared to 58.7% in those with ACLF without hyponatremia (*P* <0.001).

**Conclusions:**

The presence of hyponatremia is an independent predictive factor of survival in patients with ACLF. In cirrhosis, outcome of patients with ACLF is dependent on its association with hyponatremia.

**Electronic supplementary material:**

The online version of this article (doi:10.1186/s13054-014-0700-0) contains supplementary material, which is available to authorized users.

## Introduction

Patients with advanced cirrhosis commonly develop a functional renal impairment that render the kidney susceptible to retain sodium and solute-free water. In some patients, there is disproportionate retention of water relative to sodium, which leads to a dilutional state where water is retained out of proportion to sodium causing hyponatremia and hypoosmolality. Although hyponatremia in patients without end-stage liver disease is defined by serum sodium concentration <135 mEq/L, in cirrhosis it is defined as a serum sodium concentration of less than 130 mEq/L in the presence of ascites or edema [1-3]. In the majority of patients hyponatremia occurs in close association with an impairment of renal function and correlates with poor prognosis. In patients with cirrhosis and ascites, the five-year probability of developing hyponatremia is 37% with a 25% probability of survival at one year [4]. Hyponatremia is also an important marker of prognosis in patients with cirrhosis awaiting liver transplantation and may be associated with an increased morbidity, particularly neurological complications, and reduced survival after transplantation [5-10].

Despite the fact that there is ample data on the relationship and clinical outcomes between serum sodium, hyponatremia, and decompensated cirrhosis, there is no specific information on the frequency, characteristics, and clinical impact of hyponatremia in patients with acute-on-chronic liver failure (ACLF). ACLF is considered a syndrome that occurs in patients with chronic liver disease, with or without previously diagnosed cirrhosis, which is characterized by acute hepatic decompensation resulting in liver failure (jaundice and prolongation of the international normalized ratio (INR)) and one or more extrahepatic organ failures that is associated with increased mortality within a period of 28 days and up to three months from onset [11,12]. The chronic liver failure (CLIF) consortium recently refined the definition of ACLF on the basis of a large prospective, multicenter, observational study [13]. In the study, the overall prevalence of ACLF was 30.9% with a 90-day mortality rate of 49% [13]. Among the many variables analyzed as risk factors in relation to the six organ systems (liver, kidney, brain, coagulation, circulation and lungs) included in the modified sequential organ failure assessment (SOFA) score (CLIF-SOFA), ascites, and a high leukocyte count were found to be predictive for the development of ACLF and ACLF-associated mortality. Serum sodium or hyponatremia were independent variables that did not make it into the definition. Serum sodium (but not hyponatremia) has been included as a mortality predictor in a CLIF-Consortium score derived to predict mortality in patients with and without ACLF (CLIF-C- ACLF score) [14]. Despite these findings it is not well known if the presence of hyponatremia, a strong prognostic factor in patients with cirrhosis, influences the outcome of patients with ACLF. Therefore the aim of this analysis was to determine the specific effects of hyponatremia on the outcome of patients with ACLF.

## Methods

### Study population and data collection

This report represents an analysis of patients enroled in the Acute-on-Chronic Liver Failure (ACLF) in Cirrhosis (CANONIC) study from the CLIF consortium, which defined specific criteria for ACLF in cirrhosis [13]. ACLF was defined as an acute hepatic decompensation resulting in liver failure (jaundice and prolongation of the INR) and one or more extrahepatic organ failures in patients with chronic liver disease with or without previously diagnosed cirrhosis. In the CANONIC study, patients with cirrhosis hospitalized with an acute decompensation (AD) (ascites, gastrointestinal bleeding, hepatic encephalopathy, or bacterial infections, or any combination of these) were screened and enroled from February to September 2011 in twenty-nine University Hospitals from eight European countries. A separate Institutional Review Board approval was obtained from the original study sites (see link available at the end of the manuscript, which includes all the International Review Boards that approved the study at the various centers involved). Written informed consent was obtained from patients or their legal surrogates before inclusion. Data regarding history (including previous episodes of AD), physical examination, laboratory tests, and potential precipitating factors of ACLF were recorded. Potential precipitating factors included active alcoholism, gastrointestinal hemorrhage, bacterial infection, therapeutic paracentesis without use of intravenous albumin, transjugular intrahepatic portosystemic shunting, major surgery, and acute hepatitis.

Patients with cirrhosis admitted to the hospital with an AD were enroled in the CANONIC study according to the definition criteria of ACLF. Patients with two or more organ/system failures or those with a single renal failure (serum creatinin ≥ 2 mg/dL) or one or other organ/system failure in combination with renal insufficiency (serum creatinine between 1.5 and 1.9 mg/dL) or a 1 to 2 hepatic encephalopathy grade (West Haven scale) were found to have a poor short-term prognosis and were consequently assumed to present an ACLF episode [13].

Although hyponatremia in the general population is defined as a serum sodium ≤135 mEq/L, in cirrhosis current guidelines and consensus define it as a serum sodium level <130 mEq/L [1,2]. Thus, in this analysis hyponatremia was defined as a serum sodium level <130 mEq/L. Patients with hyponatremia at inclusion or who developed it during hospitalization were managed with fluid restriction between 1 to 1.5 liters per day according to current guidelines [2].

### Statistical analysis

Data were summarized by means of the appropriate descriptive statistics (means and standard deviation (SD) for continuous variables, frequencies and percentages for categorical parameters). Univariate analyses included Student’s *t* test or Mann-Whitney *U* test for parametric or nonparametric pairwise comparisons, respectively, and chi-square tests for categorical variables. Survival curves were estimated by means of the Kaplan-Meier method and compared through the log-rank test. The main study objective was that of assessing the relationship of ACLF and hyponatremia (and their combination) with 90-day mortality. Those risk factors showing a significant association with both ACLF and the presence of hyponatremia were taken into account as potential confounders to adjust the effect of ACLF and hyponatremia on 90-day mortality. Baseline variables associated with ACLF, hyponatremia, and 90-day mortality (that is serum creatinine, serum bilirubin or INR) were already included in the definition of ACLF, so they were not considered as potential confounders in the multivariate modeling. A proportional hazards model adjusting for these potential confounders and considering liver transplantation as a competing risk was fitted to assess the interaction between ACLF and hyponatremia. A statistically significant interaction (*P* <0.05) would lead to estimation of the effect of ACLF on mortality separately for each subset of patients with or without hyponatremia. In the absence of a significant interaction, the combination of the independent effects of ACLF and hyponatremia could be estimated through the model. Potential confounders were kept in the final model to adjust the combined effect of hyponatremia and ACLF only if they led at least to a 10% change in model coefficients estimated for the two main factors and their interaction. In all statistical comparisons, a 0.05 significance level (two-tailed) was assumed.

## Results

### Characteristics of the study population

**Figure 1:**
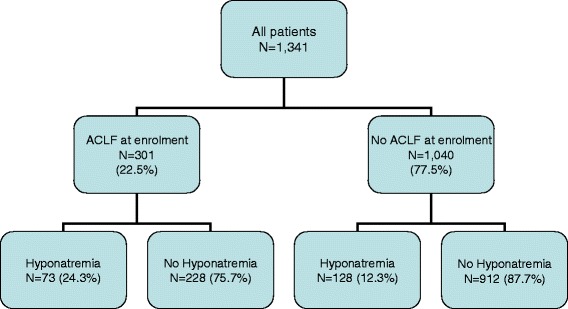
**Algorithm of all patients enroled and categorized by the presence of acute-on-chronic liver failure (ACLF) at inclusion and the subsequent development of hyponatremia after inclusion.**

The prevalence of hyponatremia in patients with and without ACLF is summarized in Figure 1. Mean serum sodium concentration in patients with hyponatremia was 125 ± 4 mEq/L compared to 137 ± 4 mEq/L in patients without hyponatremia (*P* <0.001). Patients with hyponatremia had higher frequency of bacterial infections, ascites, and hepatic encephalopathy at admission. Moreover, patients with hyponatremia showed signs of more advanced cirrhosis compared to patients without hyponatremia and, in addition, leukocyte count and C-reactive protein (CRP) levels were also higher in patients with hyponatremia (Table 1).

**Table 1:** **Characteristics of all patients according to presence of hyponatremia at study enrolment**

**Patients’ characteristics**	**Patients without hyponatremia (Na > = 130 mEq/L)**	**Patients with hyponatremia (Na <130 mEq/L)**	***P*** **value**
	**(N = 1140)**	**(N = 201)**	
Age (years)	57.2 (12.3)	56.7 (11.5)	0.60
Male sex	724 (63.5)	125 (62.2)	0.72
Alcoholic cirrhosis	550 (48)	108 (53)	0.17
Previous decompensations*	794 (72.6)	149 (77.6)	0.15
Complications at admission**			
Bacterial infections	264 (23.2)	60 (29.9)	0.04
Hepatic encephalopathy	373 (32.7)	86 (43.0)	0.005
Ascites	738 (65.1)	154 (77.4)	<0.001
Gastrointestinal bleeding	208 (18.3)	13 (6.5)	<0.001
ACLF	228 (20%)	73 (36.3%)	<0.001
*Clinical and laboratory data*			
Mean arterial pressure (mm/Hg)	84 (12.3)	80 (11.9)	<0.001
Heart rate (beats/min)	81 (16.2)	84 (16.3)	0.02
Serum bilirubin (mg/dL)	5.7 (7.4)	9.2 (9.5)	<0.001
International normalized ratio	1.6 (0.6)	1.8 (0.7)	<0.001
AST (U/L)	96 (158)	153 (282)	0.01
ALT (U/L)	56 (129)	71 (110)	0.12
GGT (U/L)	168 (278)	176 (240)	0.69
Serum creatinine (mg/dL)	1.2 (0.9)	1.7 (1.4)	<0.001
Serum sodium (mEq/L)	137 (4.1)	125 (4.3)	<0.001
Serum potassium (mEq/L)	4.1 (0.7)	4.5 (0.9)	<0.001
Leukocyte count (×10^9^ cells/L)	7.1 (4.6)	10.1 (6.0)	<0.001
Plasma C-reactive protein (mg/L)	29.9 (36.7)	36.4 (32.0)	0.03
MELD score	18.0 (7.1)	22.6 (8.3)	<0.001
Child-Pugh score	9.5 (2.1)	10.7 (2.1)	<0.001

Data are means (standard deviation (SD)) or number of patients (%). *In the three months prior to study inclusion; **between hospital admission and study inclusion. ACLF: acute-on-chronic liver failure; AST: aspartate transaminase; ALT: alanine transaminase; GGT: gamma-glutamyl transferase; MELD: model for end-stage liver disease.

### Relationship between hyponatremia and acute-on-chronic liver failure

ACLF was more prevalent in patients with hyponatremia (36.6% vs 20%, *P* <0.001) (Table 1). When patients with ACLF at inclusion were categorized according to presence or absence of hyponatremia those with hyponatremia and ACLF showed a greater impairment of liver tests (serum bilirubin and aspartate transaminase (AST) levels), higher serum creatinine, higher potassium levels, and higher model for end-stage liver disease (MELD) and Child-Pugh scores than their nonhyponatremic counterparts (Table 2). Moreover, CLIF-SOFA score, a score that evaluates the severity of cirrhosis by assessing the function of six different organs and correlates with prognosis [15], was higher in patients with ACLF and hyponatremia compared to those with ACLF without hyponatremia. Interestingly, leukocyte count was higher in patients with hyponatremia compared to that of patients without hyponatremia, despite a similar frequency of bacterial infections in the two groups (Table 2). In fact, while differences of leukocyte count in infected patients with or without hyponatremia were not significantly different, patients with hyponatremia without bacterial infection (n = 46) had significantly higher leukocyte count than patients without hyponatremia (n = 156) without bacterial infection (11,300 ± 5,700 vs. 8,800 ± 5,000, respectively, *P* = 0.0062).

**Table 2:** **Characteristics of patients with acute-on-chronic liver failure (ACLF) according to presence of hyponatremia at study inclusion**

**Patients’ characteristics**	**Patients without hyponatremia (Na > = 130 mEq/L) (N = 228)**	**Patients with hyponatremia (Na <130 mEq/L) (N = 73)**	***P*** **value**
Age (years)	56.1 (11.5)	53.7 (11.4)	0.12
Male sex	148 (64.9)	45 (61.6)	0.61
Alcoholic cirrhosis	136 (59.6)	39 (53.4)	0.54
Previous decompensations*	161 (74.9)	56 (82.4)	0.20
Complications at admission**			
Bacterial infections	72 (31.9)	27 (37.0)	0.42
Hepatic encephalopathy	130 (57.0)	44 (61.1)	0.54
Ascites	173 (76.2)	61 (85.9)	0.08
Gastrointestinal bleeding	36 (15.8)	5 (6.9)	0.05
*Clinical and laboratory data*			
Mean arterial pressure (mm/Hg)	79.7 (13.0)	77.3 (12.1)	0.15
Heart rate (beats/min)	83.5 (19.0)	83.3 (16.8)	0.94
Serum bilirubin (mg/dL)	11.0 (11.2)	14.6 (11.3)	0.02
International normalized ratio	2.1 (0.9)	2.1 (0.9)	0.90
AST (U/L)	116 (198)	233 (412)	0.03
ALT (U/L)	57 (98)	95(165)	0.10
GGT (U/L)	139 (151)	153 (194)	0.62
Serum creatinine (mg/dL)	2.2 (1.5)	2.8 (1.9)	0.01
Serum sodium (mEq/L)	136 (4.6)	125 (3.5)	<0.001
Serum potassium (mEq/L)	4.2 (0.8)	4.7 (1.1)	<0.001
Leukocyte count (×10^9^ cells/L)	9.5 (6.1)	12.1 (7.0)	0.003
Plasma C-reactive protein (mg/L)	40.9 (44.3)	42.0 (36.2)	0.86
MELD score	26.6 (7.0)	30.0 (6.6)	<0.001
Child-Pugh score	10.9 (2.1)	11.6 (2.1)	0.0341
CLIF-SOFA score***	10.1 (3.3)	11.6 (3.1)	0.0034

Data are means (standard deviation (SD)) or number of patients (%). *In the three months prior to study inclusion; **between hospital admission and study inclusion; ***CLIF-SOFA: a score that evaluates the severity of cirrhosis by assessing function of six different organs and correlates with prognosis. See reference [13]. AST: aspartate transaminase; ALT: alanine transaminase; GGT: gamma-glutamyl transferase; MELD: model for end-stage liver disease; CLIF-SOFA: chronic liver failure-sequential organ failure assessment.

### Effects of hyponatremia and ACLF on survival

At 90 days of follow-up, 264 of the 1,341 patients had died (19.7%), 961 (71.7%) were alive and 116 (8.7%) had been transplanted. The presence of hyponatremia (either at inclusion or during hospitalization) was a predictive factor of survival both in patients with and without ACLF. Several factors measured at study enrolment were associated with both hyponatremia and/or ACLF (Tables 1 and 2) and, at the same time, some were found to be predictors of 90-day mortality: age, presence of ascites and bacterial infections, mean arterial pressure, heart rate, serum potassium and white cell count. All these variables were taken into account as potential confounders for adjusted estimates of the effects of ACLF and hyponatremia on mortality.

**Figure 2:**
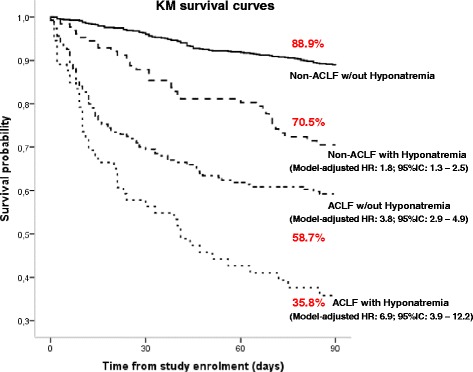
**Transplant-free survival curves in patients with and without acute-on-chronic liver failure (ACLF) according to the presence of hyponatremia at inclusion.** CLIF: chronic liver failure; CLIF-SOFA: chronic liver failure-sequential organ failure assessment; HR: hazard ratio; MELD: model for end-stage liver disease.

The competing-risks proportional hazards model was first fitted including all the potential confounders selected in the univariate analyses, hyponatremia, ACLF and the interaction of the two main factors (Table 3). The interaction between hyponatremia and ACLF was not statistically significant (*P* = 0.53), thus the effects of hyponatremia and ACLF were assumed as independent and adjusted for the potential confounders in order to obtain the final model estimates. After adjusting for confounding variables, hyponatremia without ACLF was found to nearly double the risk of dying at 90 days, while for patients with both ACLF and hyponatremia the relative risk was nearly seven times higher than for patients without either factor (Table 3). The corresponding survival curves of patients with and without ACLF according to the presence of hyponatremia at inclusion are shown in Figure 2. In patients without ACLF, the presence of hyponatremia was associated with a poor prognosis. In fact, patients with hyponatremia without ACLF had a 90-day survival probability of 70.5% compared to 88.9% in patients without ACLF and without hyponatremia (*P* <0.001). Moreover, the presence of hyponatremia was associated with even a poorer prognosis in patients with ACLF. Patients with ACLF without hyponatremia had a 90-day survival probability of 58.7%, compared to only 35.8% in patients with ACLF and hyponatremia (*P* = 0.001). Similar differences in survival were observed when both patients with hyponatremia at inclusion and during hospitalization were considered (Figure S1 in Additional file 1).

**Table 3:** **Assessment of the interaction between acute-on-chronic liver failure (ACLF) and hyponatremia at inclusion and estimation of the risk of 90-day mortality adjusted by potential confounding factors**

**Assessment of ACLF-by-hyponatremia interaction**			**Estimate of the independent effect of ACLF and hyponatremia**		
**Parameter**	Hazard ratio (95% CI)*	***P*** **value**	**Parameter**	Hazard ratio (95% CI)*	***P*** **value**
ACLF at study enrolment	3.99 (2.92-5.44)	<0.001	ACLF at study enrolment	3.78 (2.90-4.93)	<0.0001
Hyponatremia at study enrolment	2.00 (1.33-3.02)	0.001	Hyponatremia at study enrolment	1.81 (1.33-2.47)	0.0002
			*Combination of independent effects:*		
Interaction ACLF-by-hyponatremia	0.83 (0.47-1.48)	0.5300	ACLF/hyponatremia vs. no ACLF/no hyponatremia	6.85 (3.85-12.19)	<0.0001

*Hazard ratio estimates from a competing-risks proportional hazards model, adjusting for age, presence of ascites, presence of bacterial infections, white cell count, heart rate and serum potassium at study enrolment. CI: confidence interval.

## Discussion

This study represents an extensive assessment of the influence of hyponatremia in patients with ACLF. ACLF is considered a distinct entity apart from decompensated cirrhosis; it is defined as an abrupt hepatic deterioration in patients with pre-existing chronic liver disease, which is usually related to a precipitating event and associated with increased mortality at three months due to multisystem organ failure [11-13]. So despite the fact that hyponatremia is a well-recognized complication of patients with advanced cirrhosis, a specific analysis of hyponatremia in patients with ACLF had so far not been reported. The investigation of this relationship is clinically relevant, given the important physiological effects of low serum sodium levels and the well-demonstrated relationship between hyponatremia and survival in the global population of patients with decompensated cirrhosis [4-7,15-19]. In this analysis, we have shown that the presence of hyponatremia in patients with ACLF influences outcome. Interestingly, both variables (hyponatremia and ACLF) independently affect this outcome. Thus the presence of hyponatremia in a patient without ACLF significantly increases the risk of dying at 90 days, but when patients with both (ACLF and hyponatremia) are compared to those without either (ACLF and hyponatremia) then there is an even higher risk of dying at 90 days. These findings, to our knowledge, have not been reported in this subset of patients.

As expected, and in keeping with previous studies, the presence of hyponatremia was associated with increased three-month mortality [15-19]. However, a relevant observation of this study was that the prognosis of patients with ACLF was strongly dependent on the presence or absence of concomitant hyponatremia. In fact, patients with ACLF plus hyponatremia had very low three-month survival expectancy compared to that of patients with ACLF without hyponatremia (35.8% vs. 58.7%, respectively; *P* <0.001). Similar findings were also observed if patients who developed ACLF during hospitalization were taken into consideration (44.5% vs. 61.5%, respectively; *P* <0.001). On the other hand, in patients without ACLF, the presence or absence of hyponatremia also influenced prognosis, in such a way that the group of patients without ACLF without hyponatremia had an excellent three-month survival, near 90%, much better than that of patients without ACLF but with hyponatremia.

For many years hyponatremia in patients with cirrhosis has been clearly described as an independent risk factor for mortality [4-7]. The mechanisms that drive this poor prognosis are likely related to its occurrence along with other complications of cirrhosis. In a survey study of 997 cirrhotic patients, Angeli *et al*. demonstrated a prevalence of serum sodium ≤130 mmol/L of 21.6% [20]. This patient subgroup had a significantly higher incidence of hepatic encephalopathy (odds ratio (OR) 3.40; 2.35 to 4.92), hepatorenal syndrome (OR 3.45; 2.04 to 5.82), and spontaneous bacterial peritonitis (OR 2.36; 1.41 to 3.93). It is estimated that patients with cirrhosis and hyponatremia have a 25-50% probability of survival at one year and 23% at five years [4,5]. In contrast to what occurs in cirrhosis in patients with ACLF, hyponatremia portends a 35% probability of survival at three months. The mechanistic reason as to why such as difference exists has not been properly assessed, but it is known that in ACLF increasing organ failures certainly drive prognosis, whereas in decompensated cirrhosis this does not necessarily occur.

Although not a primary endpoint, we found an interesting and previously unreported association between hyponatremia and leukocyte count in these patients. These findings do not reflect the aim of the study, which was to focus on the outcome of patients with ACLF and hyponatremia. Nonetheless two variables, ACLF and leukocyte count, were associated with the presence of hyponatremia in this large cohort of patients. This relationship between leukocyte count and hyponatremia appeared to be independent from bacterial infections, because among patients without bacterial infections, those with hyponatremia had significantly higher leukocyte count than that of patients without hyponatremia. This relationship is intriguing and may have pathophysiological relevance. Alternatively, it could also be possible that cytokines may interfere directly in kidney water metabolism, causing an impaired water excretion as suggested in other disease states [21,22]. In patients without cirrhosis, development of hyponatremia has been associated with inflammatory diseases such meningitis, pneumonia, tuberculosis, encephalitis, human immunodeficiency virus infection, and malaria [23,24]. However, this needs to be properly studied in patients with cirrhosis and also in those with ACLF.

## Conclusions

The results of the current study show that there is an important association between hyponatremia and ACLF. Hyponatremia is not only a prognostic marker in patients with ACLF, but influences the outcome of these patients. Mortality rates are clearly different among patients with ACLF with and without hyponatremia. In patients with ACLF prognosis is clearly dependent on its association with hyponatremia.

## Additional file

Additional file 1: Figure S1. Transplant-free survival curves in patients with and without ACLF according to the presence of hyponatremia during hospitalization.
