# Supplementary material for: Hyponatremia influences the outcome of patients with acute-on-chronic liver failure: an analysis of the CANONIC study
Source: Crit Care. 2014 Dec 13;18(6):700. doi: 10.1186/s13054-014-0700-0 (PMC4280050; doi:10.1186/s13054-014-0700-0)
Supplement: Additional file 1: Figure S1. — Transplant-free survival curves in patients with and without ACLF according to the presence of hyponatremia during hospitalization. [file 13054_2014_700_MOESM1_ESM.ppt]

## Slide 1
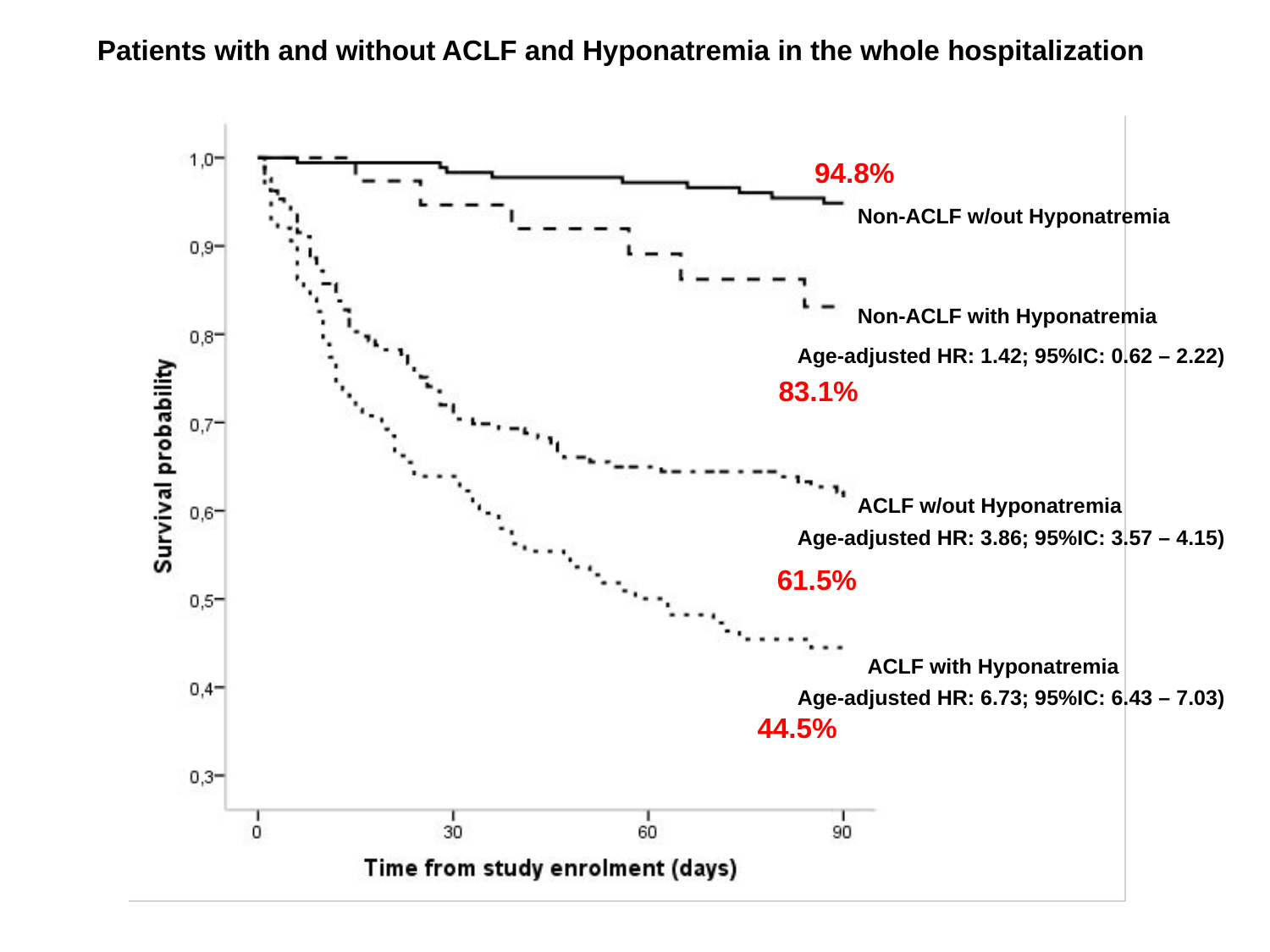

Patients with and without ACLF and Hyponatremia in the whole hospitalization
94.8%
Non-ACLF w/out Hyponatremia
Non-ACLF with Hyponatremia
Age-adjusted HR: 1.42; 95%IC: 0.62 – 2.22)
83.1%
ACLF w/out Hyponatremia
Age-adjusted HR: 3.86; 95%IC: 3.57 – 4.15)
61.5%
ACLF with Hyponatremia
Age-adjusted HR: 6.73; 95%IC: 6.43 – 7.03)
44.5%
